# Expression levels of ERCC1 and RRM1 mRNA and clinical outcome of advanced non-small cell lung cancer

**DOI:** 10.12669/pjms.295.3812

**Published:** 2013

**Authors:** Bi Jian-wei, Mao Yi-min, Sun Yu-xia, Liu Shi-qing

**Affiliations:** 1 Bi Jian-wei, Respiratory and Critical Care Medicine, the First Affiliated Hospital, Henan University of Science and Technology, Luoyang, China.; 2 Mao Yi-min, Respiratory and Critical Care Medicine, the First Affiliated Hospital, Henan University of Science and Technology, Luoyang, China.; 3 Sun Yu-xia, Respiratory and Critical Care Medicine, the First Affiliated Hospital, Henan University of Science and Technology, Luoyang, China.; 4 Liu Shi-qing, Preventive Medicine, the Affiliated Qianfoshan Hospital, Shandong University, Ji’nan, China.

**Keywords:** ERCC1, RRM1, mRNA, Outcome, Advanced non-small cell lung cancer

## Abstract

***Objective: ***We conducted a study to understand the genetic effect of ERCC1 and RRM1 on the chemotherapy response and clinical outcome of Non-Small Cell Lung Cancer (NSCLC).

***Methods:*** The relative cDNA quantification for ERCC1 and RRM1 was conducted using a fluorescence-based real-time detection method among 294 NSCLC patients.

***Results:*** Compared with the internal reference gene β-action, the median levels of ERCC1 and RRM1 expression were 2.43×10^-2^ and 0.11×10^-2^, respectively. Our study showed response to platinum-containing regimen chemotherapy was high in those with high ERCC1 expression, and the OR (95% CI) were 1.73(1.06-2.81). An apparently high response to chemotherapy was decreased when patients were carrying both high expression of ERCC1 and RRM1, with OR (95% CI) of 2.57(1.21-4.90). Patients with high expression of ERCC1 were associated with a longer OS by multivariate Cox proportional hazards model. Moreover, those carrying both high levels of ERCC1 and RRM1 were seem to have a longer OS when compared with those with low expression (HR=0.31, 95% CI=0.13-0.62 for OS).

***Conclusion:*** This observation could be used in personalized chemotherapy, increase the response rate and prolonged survival time, and could encourage to explore the predictive value of other genes.

## INTRODUCTION

Lung cancer is a common malignancy with high-incidence and mortality, which is reported to be the highest fate cancer in China and worldwide.^1^ Non-small cell lung cancer accounted for almost 85% of all cases, and most of them have been in advanced stage when they were diagnosed. Chemotherapy is one of the major treatment options for advanced NSCLC, customizing chemotherapy by biomarkers have improve the clinical outcome in patients with NSCLC. Cisplatin, a chemotherapy regime, has a critical function in the treatment of advanced NSCLC.

Bulky DNA adducts by cisplatin are mainly repaired by the mechanism of nucleotide excision repair pathway (NER).^2^^,^^3^ During the NER pathway, the proteins of the excision repair cross complementing 1 (ERCC1) and ribonucleotide reductase subunit M1(RRM1) play an important role in this pathway.^2^^,^^3^ ERCC1 is a DNA damage repair gene and encodes the 5’endonuclease during the NER pathway, and an increase in ERCC1 expression is likely to cause the cisplatin resistance phenotype. Cisplatin causes cytotoxicity of cancer cells through forming adducts that result in DNA cross-links. RRM1 located on chromosome segment 11p15.5 usually showed a frequent loss of heterozygosis in NSCLC. High levels of RRM1 levels are reported to be the predictive biomarkers for the response to chemotherapy.

Recently, DNA repair genes have been confirmed as predictive markers for the chemotherapy treatment and clinical outcome of patients with NSCLC.^4^^,^^5^ Better understanding of genetic effect on the chemotherapy response could offer important information for customizing chemotherapy treatment. ^4^^,^^5^

## METHODS

***Subjects:*** A total of 326 patients who were histologically confirmed advanced NSCLC (inoperable stage IIIB and IV NSCLC) were enrolled into this study between January 2009 and January 2010. All the biopsy samples were collected either from bronchoscopic or fine needle aspiration biopsies. Finally, 294 patients agreed to participate into the study, with a participation rate of 90.2%. All the patients received platinum-based combination chemotherapy at the First Affiliated Hospital of Henan University of Science and Technology. Patients with other malignant cancer history for five year or who had pregnancy or lactation, cardiopulmonary insufficiency, serious cardiovascular disease and serious infection as well as severe malnutrition were excluded. The demographic and clinical characteristics were collected by medical records and a self-designed questionnaire.

***Study design: ***All patients received platinum-based chemotherapy, including gemcitabine, vinorelbine or paclitaxel. The intravenous dosage of cisplain was 75mg/m^2^, and carboplatin and gemcitabine 1,000 mg/m^2^ on day one and eight, vinorelbine 25mg/m^2^ on day one and day eight for a maximum of four cycles, and the treatments were suspended until disease progression or unacceptable toxicity. If patients showed three grades of non-hematology toxicity, four grades of hematology toxicity, febrile neutropenia or infection, the dosage of chemotherapeutic drug was reduced by 25% of the next cycle. When the chemotherapy was finished, the patients were followed up every month by telephone up to death or the end of study. The response to chemotherapy was classified by RECIST criteria.^6^ We also evaluated the overall survival (OS) and progression-free survival (PFS). The OS was defined from the start of therapy to the date of death, and PFS was defined from the start of therapy to the date of progression or death without progression. All patients gave informed consent before enrolling into the study, and our study was approved by the Ethics Committee of the First Affiliated Hospital of Henan University of Science and Technology.

All patients provided 5 ml peripheral venous blood samples before they received the first cycle of chemotherapy. The total RNA was extracted using an EZNA Blood RNA Mini Kit (Omega, Berkeley, CA, US) and dissolved in water according to manufacturer’s instructions. The isolated RNA samples were stored at -70ºC, and complementary DNA was synthesized using a Reverse Transcription System (Promega, Madison, US) during one week. Then the produced cDNA was stored at -20ºC.

The relative cDNA quantification for ERCC1 and RRM1 was conducted using a fluorescence-based, real-time detection method, and β-actin was used as a reference gene. All primers were designed using online Primer Premier 5.0 software (http://www.premierbiosoft.com/primerdesign). The primers of ERCC1 and RRM1 were as follows: ERCC1: 5’-CTG GGA ATT TGG CGA CGT AA-3’(forward primer), 5’-ATG GAT GTA GTC TGG GTG CAG-3’(reverse primer); RRM1: 5’-AAG CTG GAA AAG ACC CTG CC-3’(forward primer) and 5’-CTC GGG TGA GGA ACA GTC CA-3’(reverse primer). The PCR conditions started with a denaturation at 95ºC for 10 minutes, followed by 40 cycles of denaturation at 95ºC for 20s, and finally with annealing at 60ºC for 60s. When comparing the threshold cycle with the standard curve, the relative amount of cRNA was determined, and standardized amount of them was determined by using the β-actin amount

***Statistical analysis: ***Continuous variables were showed as mean ± SD and categorical variables were presented as number of subjects (%). Patients who showed complete response (CR) or partial response (PR) were defined as ‘‘responders’’, and those with stable disease (SD) or progressive disease were regarded as ‘‘non-responders’’. Odds ratios (OR) and their corresponding 95% confidence intervals (CI) were used to assess the association between gene expression and response. Overall survival (OS) was measured from the start of chemotherapy to the date of death from any causes or last clinical follow-up. The association between the different mRNA expression and survival was estimated using hazard ratios (HR) their confidence intervals (CI) from multivariate Cox proportional hazards model. Survival distributions were estimated by using the Kaplan-Meier method and assessed using the log-rank test. *P* value less than 0.05 was considered to be significant. All tests were two-sided and analyzed by SPSS 11.0 software.

## RESULTS

***Patient characteristics: ***Clinical data of 294 patients is summarized in Table-I. The median age of all patients was 61.3 years and range from 27.4 to 80.5 years. Among them, 68.7% were men, and 31.3% were women. 42.5% were stage IV NSCLC, and 34.4% were adenocarcinoma. All patients received platinum-based chemotherapy, and were followed-up until December 2012. During the follow-up period, 11(3.7%) patients lost to follow-up and 131(44.5%) patients died.

**Table-I:** Characteristics of included patients

***Characteristics***	***Number***	***Percentage (%)***
Median age (years)	61.3(27.4-80.5)	
Gender		
Male	202	68.7
Female	92	31.3
Smoking status		
No	197	67.1
Yes	97	32.9
Stage		
IIIB	169	57.5
IV	125	42.5
Histopathology		
Adenocarcinoma	101	34.4
Squamous	193	65.6
Response to chemotherapy		
CR or PR	152	51.7
SD	142	48.3

β-action was used as an internal reference gene. The cut points of the ERCC1 and RRM1 expression levels were determined using the median expression levels of all the patients. When compared with the internal reference gene β-action, the median levels of ERCC1 and RRM1 expression were 2.43×10^-2^ and 0.11×10^-2^, respectively. The expression of ERCC1 and RRM1 were further divided into high and low expression according to the median levels (Table-II). The results showed response to platinum-containing regimen chemotherapy was high in those with high ERCC1 expression, and the OR (95% CI) were 1.73(1.06-2.81). Patients with high expression of RRM1 benefited more from chemotherapy, but a non-significant OR was found (OR=1.37, 95% CI=0.83-2.26). Moreover, we found an apparently high response to chemotherapy when patients carrying both high expression of ERCC1 and RRM1, with OR (95% CI) of 2.57(1.21-4.90).

Patients with high expression of ERCC1 were associated with a longer OS than those with low expression (11.7 vs 17.4 months, P=0.03 for OS), and the HR (95% CI) was 0.63(0.35-0.88) by multivariate Cox proportional hazards model (Table-III). Those carrying both high levels of ERCC1 and RRM1 were seem to have a longer OS when compared with those with low expression (18.7 vs 10.6 months, P=0.028 for OS), and a significant strong HR (95% CI) was found (HR=0.31, 95% CI=0.13-0.62 for OS).

**Table-II:** Association between ERCC1 and RRM1 and response to chemotherapy

*Expression level*	*N*	*%*	*Responders*	*%*	*Non-responders*	*%*	*OR(95% CI)*	*P value*
Low ERCC1	145	49.3	65	42.8	80	56.3	-	-
High ERCC1	149	50.7	87	57.2	62	43.7	1.73(1.06-2.81)	0.02
Low RRM1	109	37.2	51	33.6	58	41.1	-	-
High RRM1	185	62.8	101	66.4	84	58.9	1.37(0.83-2.26)	0.19
Low ERCC1/Low RRM1	61	20.7	23	15.1	38	26.8	-	-
High ERCC1/ Low RRM1	48	16.3	28	18.4	20	14.1	2.32(0.98-5.39)	0.05
Low ERCC1/ High RRM1	84	28.6	42	27.6	42	29.6	1.65(0.81-3.43)	0.14
High ERCC1/ High RRM1	101	34.4	59	38.8	42	29.6	2.57(1.21-4.90)	0.01

**Table-III:** Association between ERCC1 and RRM1 expression and survival of NSCLC

*Expression level*	*Overall survival*		
	*Median survival (95% CI, months)*	*Log-rank P*	*HR (95% CI)*
Low ERCC1	11.7(2.6-15.3)	-	-
High ERCC1	17.4(4.3-22.6)	0.03	0.63(0.35-0.88)
Low RRM1	13.5(3.2-16.4)	-	-
High RRM1	15.4(3.6-18.4)	0.08	0.77(0.42-1.54)
Low ERCC1/Low RRM1	10.6(3.0-15.7)	-	-
High ERCC1/ Low RRM1	16.2(3.2-18.9)	0.06	0.67(0.36-1.03)
Low ERCC1/ High RRM1	12.5(3.1-15.8)	0.54	0.85(0.61-1.56)
High ERCC1/ High RRM1	18.7(4.5-23.1)	0.004	0.31(0.13-0.62)

## DISCUSSION

The standard first-line chemotherapies, such as paclitaxel, gemcitabine, docetaxel, or vinorelbine, is usually used in combination with the platinum chemotherapy compound, and the treatment has become the main adjuvant treatment for various cancers, including NSCLC, bladder cancer and pancreatic cancer.^7^ Individualized chemotherapy according to reliable molecular prognostic and predictive markers may play an important role in the clinical outcome of cancer patients. Previous clinical studies have investigated the role of RRM1 and ERCC1 expression levels in the chemotherapy resistance among NSCLC.^8^^-^^10^ The present study suggests that high expression of RRM1 was associated with longer survival time and better response to chemotherapy when compared with low expression. Moreover, the association is stronger among those carrying both high expression of RRM1 and ERCC1. 

ERCC1 located on chromosome segment 19q13.2-3 usually showed a frequent loss of heterozygosis in NSCLC, and its expression showed different response to gemcitabine chemohterapy. Previous studies indicated that ERCC1 level could benefit significantly from cisplatin, oxaliplatin or gemcitabine in cancer patients.^9^^,^^11^^,^^12^ A study conducted in China with 130 advanced NSCLC indicated a significantly longer survival time was seen in patients with high ERCC1 expression compared to patients with low ERCC1, but it did not find the ERCC1 expression could benefit more from platinum-based chemotherapy.^11^ Another study conducted in Czech Republic reported the a significant association between mRNA levels of ERCC1, and it found a significantly longer expression of ERCC1 was related to a longer disease-free interval in advanced NSCLC patients.^9^ In our study, we found high ERCC1 expression gained a significant higher response rate, progression-free survival and overall survival than those with low expression, which is in line with previous studies. However, two studies reported the ERCC1 protein expression was not associated with response to platinum-based chemotherapy or clinical outcome of NSCLC.^13^^-^^15^ The inconsistency of these results may be induced by differences in population background, selection of control, sample size or by chance. Further large sample studies are warranted to clarify their association.

RRM1 has role in DNA repair systems like ERCC1 does. Ren SX et al reported that chemotherapy customized in terms of RRM1 expression levels is associated with higher response rate and longer PFS and OS in patients who received chemotherapy.^16^ Another study which was also conducted in China the expression of RRM1 in tumor tissues and peripheral blood lymphocytes is closely correlated with the response to chemotherapy and prognosis of patients with advanced NSCLC treated with adjuvant chemotherapy.^15^ In our study, we found a strong association between high ERCC1 and RRM1 expression and response rate to chemotherapy and survival outcomes, which indicated the interaction between ERCC1 and RRM1 genes. 

In conclusion, the present study suggested that RRM1 and ERCC1 may be a predictive and prognostic indicator in advanced NSCLCL patients receiving chemotherapy. This observation could be used in personalized chemotherapy and increase the response rate and prolonged survival time, and could encourage us to explore the predictive value of other genes.
